# Impact of urinary PAHs on psoriasis risk in U.S. adults: Insights from NHANES

**DOI:** 10.1371/journal.pone.0314964

**Published:** 2024-12-05

**Authors:** Jiang-Hui Li, Xiao-Ning Yan, Jia-Ying Fu, Hao-Yuan Hu

**Affiliations:** 1 The First Clinical Medical college, Shaanxi University of Chinese Medicine, Xianyang, China; 2 Department of Dermatology, Shaanxi Hospital of Traditional Chinese Medicine, Xi’an, China; Center for Research and Technology Transfer, VIET NAM

## Abstract

**Objective:**

Exposure to environmental pollutants is increasingly recognized as a risk factor for the development of psoriasis. Polycyclic aromatic hydrocarbons (PAHs) are ubiquitous in the air and might induce reactions such as oxidative stress. Nevertheless, it is still unclear if PAHs have any influence on the prevalence of psoriasis over the entire population of the United States. The objective of this study was to assess the association between urine PAHs and psoriasis.

**Methods:**

The research included 3,673 individuals aged 20 years or older who participated in the 2003–2006 and 2009–2012 National Health and Nutrition Examination Surveys (NHANES). We employed logistic regression models to evaluate the relationship between levels of urine PAH metabolites and psoriasis and smoothed curve fitting to illustrate the concentration-response relationship. Additionally, subgroup and interaction analyses were conducted to elucidate these associations. Furthermore, we employed weighted quartile sum (WQS) regressions to examine the distinct effects of individual and mixed urine PAH metabolites on psoriasis. However, it is important to note that the NHANES sample may be subject to selectivity and self-reporting bias, which may influence the data’ generalisability.

**Results:**

We observed that the highest tertiles of 2-NAP and 2-FLU had a 63% (95% CI 1.02, 2.61) and 83% (95% CI 1.14, 2.96) higher odds of association with psoriasis prevalence, respectively. Meanwhile, tertile 2 and tertile 3 of 3-PHE were also significantly associated with psoriasis, with higher odds of 65% (95% CI 1.01, 2.69) and 14% (95% CI 1.17, 3.00), respectively. The subgroup analyses revealed a significant correlation between urine PAH metabolites and the odds of psoriasis in specific groups, including males, aged 40–60 years, with a BMI > 30, and those with hyperlipidemia. In the WQS model, a positive association was found between the combination of urine PAH metabolites and psoriasis (OR 1.43, 95% CI 1.11, 1.84), with 2-FLU being the most prevalent component across all mixtures (0.297).

**Conclusions:**

Our findings indicate a significant association between urine PAH metabolites and the odds of psoriasis prevalence in adults. Among these metabolites, 2-FLU demonstrated the most prominent impact. Controlling PAH exposure, as an important strategy for minimizing exposure to environmental contaminants and lowering the risk of psoriasis, is critical for raising public knowledge about environmental health and preserving public health.

## Introduction

Psoriasis is a common, chronic inflammatory skin condition that impacts approximately 2–3 percent of the world’s population [1]. Psoriasis is distinguished by well-defined red patches covered in silvery-white scales. These patches often appear symmetrically on the scalp or trunk and can cause varying degrees of itching [2–4]. Psoriasis has detrimental effects on both the skin and overall quality of life. It also decreases life expectancy and leads to several other health conditions, including psoriatic arthritis, diabetes, metabolic syndrome, and cardiovascular disease [5]. The precise cause of psoriasis is not completely understood. However, it is believed to be influenced by various variables including genetic predisposition, immunological dysregulation, and possible environmental influences [6].

Humans are exposed to an extensive range of pollutants originating from both human activities and natural sources. According to the World Health Organization (WHO), over 4.2 million individuals perish each year due to the effects of air pollution in the environment [7]. Polycyclic aromatic hydrocarbons (PAHs) are a significant part of the air pollutants found in the environment. They are produced and emitted into the air during the procedure of burning coal and oil, transportation, incinerating garbage, and steaming meals [8]. People’s lives and environmental exposure patterns have altered substantially as the world grows more urbanized and pollution levels rise. PAHs can be identified in water, soil, and air in trace amounts, and they are regularly encountered by humans through inhalation, ingestion, and skin contact in their daily environment. Furthermore, changes in environmental policy may have an impact on PAH emission and control, with potentially far-reaching consequences for public health. The impact of PAHs on the human body is mediated by intricate pathways, primarily through the Aryl hydrocarbon Receptor (AhR) pathway, oxidative stress pathway, and associated immunological issues [9]. Recent studies have shown that PAHs may increase the risk of several disorders, such as cardiovascular diseases [10], lung and skin cancers [11], hyperlipidemia [12], endometriosis [13], and neuropsychiatric abnormalities [14].

Prior NHANES studies demonstrated that exposure to elevated air pollution heightens the risk of inflammatory skin diseases such as psoriasis [15, 16]. However, they were unable to examine urinary concentrations for specific PAHs and the potential correlation between various subgroups and psoriasis. Hence, understanding the link between PAH exposure and psoriasis is therefore critical in today’s context. Exploring their relationship can help us identify high-risk communities and provide data to politicians in order to enhance environmental quality and population health. This study made use of the NHANES dataset, which contains a wealth of information on the health status, nutritional intake, and environmental exposures of the United States population and serves as an important foundation for analyzing the relationship between PAH and the risk of psoriasis.

## Methods

### Data sources and participant information

**Fig 1:**
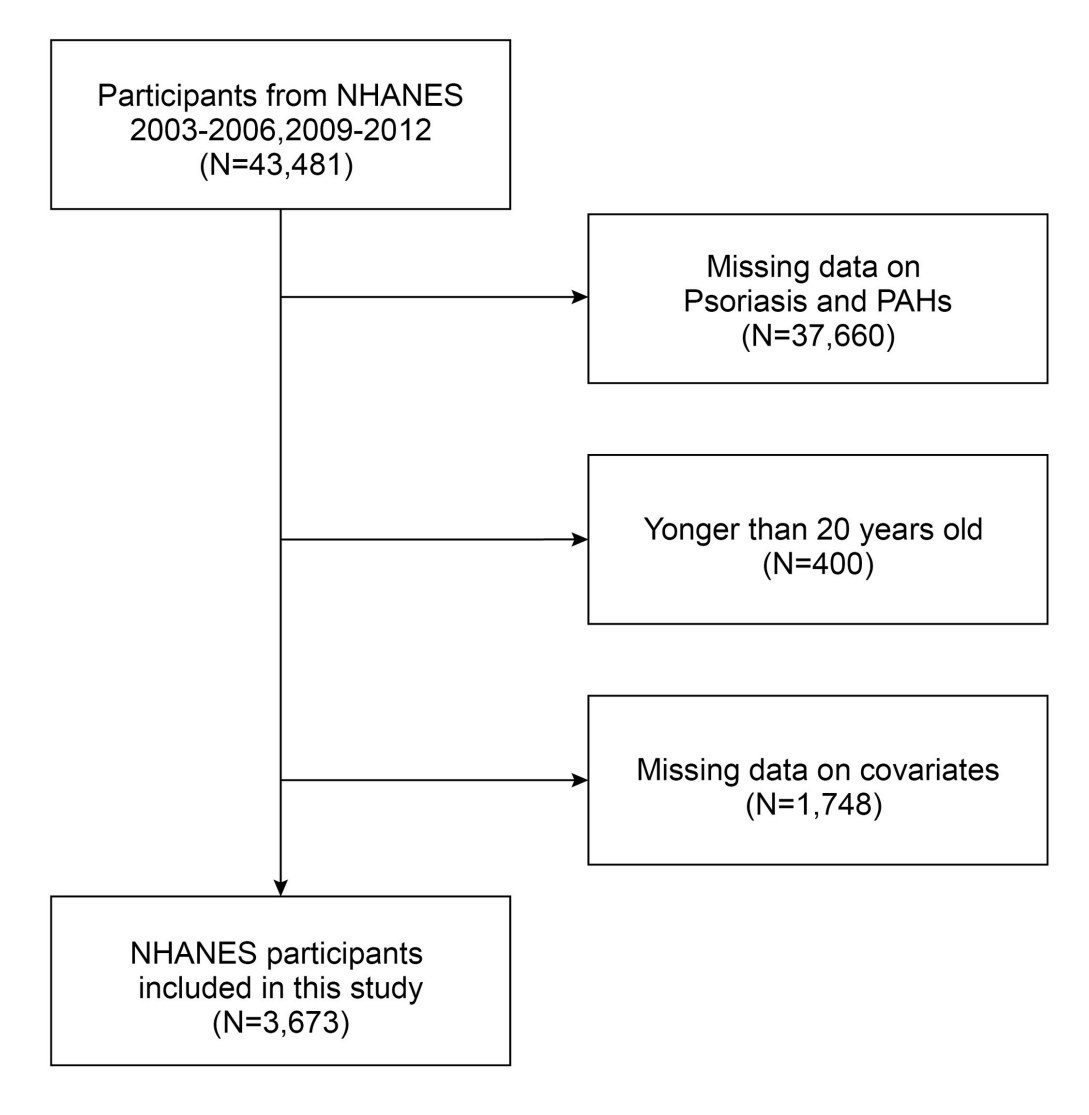
Screening flowchart for eligible participants.

All data are accessible on the NHANES website, a nationally representative cross-sectional survey of diet and health status in the United States. Each participant in the study provided their affirmative consent, and the NHANES protocol underwent a comprehensive evaluation and received permission from the National Center for Health Statistics Research Ethics Review Board. The study utilized data from the NHANES cycles of 2003–2006 and 2009–2012 to investigate the association between urine PAH concentrations and the odds of psoriasis. At the beginning, a total of 43,481 participants were included in the analysis across the four study cycles. The final study group comprised 3,673 participants after excluding 37,660 individuals without data on both urinary PAHs metabolites and psoriasis, as well as 400 participants under the age of 20 and 1,748 with incomplete information on education level, poverty income ratio (PIR), BMI, alcohol consumption, hypertension, diabetes, and hyperlipidemia. Fig 1 illustrates the precise criteria used to determine whether participants should be included or excluded from this study.

### Urinary PAHs metabolite measurement

Individuals who were at least 6 years old were eligible to take part in the test. We conducted a study on the quantification of eight urinary PAH metabolites: 1- Hydroxynaphthalene (NAP), 2-NAP, 2-Hydroxyfluorene (FLU), 3-FLU, 1-Hydroxyphenanthrene (PHE), 2-PHE, 3-PHE, 1-Hydroxypyrene(PYR). The results of this study were obtained in 2003–2006 based on capillary gas chromatography with high-resolution mass spectrometry (GC-HRMS), 2009–2012 based on isotope dilution capillary gas chromatography-tandem mass spectrometry (GC-MS/MS). Proficient technicians collected urine samples, which were then stored at a temperature of -20°C. The NHANES webpage offers comprehensive information regarding measurements. In accordance with NHANES laboratory guidelines, values below the lower limit of detection (LLOD) were computed as LLOD/√2. The concentration of PAHs (ng/L) in urine was normalized by dividing it by the urinary creatinine level (mg/dL) multiplied by 0.01. This normalization allows the data to be reported as nanograms per gram of creatinine and helps to minimize errors in the data. In order to facilitate analysis, the data were logarithmically converted.

### Definition of psoriasis

The diagnosis of psoriasis was ascertained through self-report on health questionnaires based on participants’ responses to the following question, “{Have you/Has SP} ever been told by a doctor or other health care professional that {you/s/he} had psoriasis?". If the subject answered "Yes," they were diagnosed with psoriasis.

### Covariables

According to prior research on the prevalence of PAH and psoriasis [17, 18], confounding factors such as an individual’s health management, socioeconomic status, and environmental exposures may influence the results. Thus, we chose the following variables as covariates, including age, gender, race, education level, marital status, PIR, BMI, alcohol consumption, serum cotinine levels, and the presence of hypertension, diabetes mellitus, and hyperlipidemia. The participants’ marital status was categorized as either unmarried or married. The poverty income ratio was categorized as either 0–4.99 or ≥5. The participants’ BMI was categorized into three groups: normal weight (<24.9 kg/m^2^), overweight (24.9 kg/m^2^-30 kg/m^2^), and obesity (>30 kg/m^2^) [19]. Hypertension was diagnosed based on systolic/diastolic blood pressure readings ≥ 130/80 mmHg, self-reported clinician diagnosis, or self-reported use of hypertension medications [20]. Glycosylated hemoglobin ≥6.5%, fasting blood glucose ≥ 7.0 mmol/L, self-reported medical diagnosis, or self-reported insulin use were used to identify diabetes [21]. The National Cholesterol Education Program (NCEP) Adult Treatment Panel III established the following criteria for hyperlipidemia: LDL-C ≥130 mg/dL, TC ≥200 mg/dL, TG ≥150 mg/dL, or HDL-C ≤50 mg/dL in women and ≤40 mg/dL in men [22]. Hyperlipidemia was also deemed to exist in those on anti-hyperlipidemic medications.

### Statistical analysis

Continuous variables are represented as mean ± standard deviation (SD), while categorical data is stated as percentage (%). The participants were classified into two distinct groups: those with psoriasis and those without psoriasis. The baseline characteristics of the population were assessed by employing linear regression models and chi-square tests, conducted separately for each group. Pearson correlation analysis was utilized to determine the correlation coefficients between PAH metabolites. A multivariate logistic regression model was created to explain the relationship between the eight urine PAH metabolites and the risk of psoriasis. In this analysis, we categorized the eight PAHs into three groups based on their distribution across tertiles: tertile 1, tertile 2, and tertile 3. Model 1 did not include any covariables. Model 2 adjusted for age, sex, and race, while Model 3 adjusted for all covariates, including age, sex, race, education level, marital status, PIR, BMI, alcohol use, presence of hypertension, diabetes, hyperlipidemia, and serum cotinine levels. The strength of the link was measured by calculating odds ratios (ORs) and 95% confidence intervals (CIs). We utilized smoothed curve-fitting to depict the connection between urine PAH metabolite concentrations and the risk of acquiring psoriasis. Furthermore, we conducted an interaction analysis to evaluate the correlation between levels of urine PAH metabolites and the odds of psoriasis in specific subgroups, with the aim of identifying potential risk factors. Lastly, we used the "gWQS" R package to execute a WQS regression in order to evaluate the impact of combining urine PAH metabolites on the prevalence of psoriasis. The cumulative impact of mixed urine PAH metabolites on psoriasis was demonstrated by this study, and the proportionate contribution of each exposure variable to the total effect was ascertained. The statistical analyses were conducted using R 4.3.1 and EmpowerStats 4.2 software. All tests were conducted using a two-sided approach, and a *P*-value <0.05 is considered to be significant.

### Ethics statement

This study was based on a publicly available database and did not require ethical approval or consent. Studies involving humans were approved by the National Center for Health Statistics (NCHS). The studies were conducted in accordance with local legislation and institutional requirements. Participants provided written informed consent to participate in this study.

## Results

### Participant characteristics

The individuals’ characteristics are presented in Table 1. The study encompassed a total of 3,673 participants, ranging in age from 20 to 80. These subjects provided information about their psoriasis status and also underwent tests to measure urinary PAHs metabolites. Out of the entire participant group, 127 (3.5%) individuals were diagnosed with psoriasis, whereas the remaining 3,546 (96.5%) people did not have psoriasis. The mean age of the participants was 48.28 ± 16.58 years, with 51.18% being male. The majority of the participants were non-Hispanic whites (47.54%), held an AA or Some College degree (30.85%), and were unmarried (75.52%). Significant differences were observed between the psoriasis groups in terms of race, the prevalence of hyperlipidemia, serum cotinine levels, and urinary PAH metabolites including 2-NAP, 2-FLU, 3-FLU, 1-PHE, 2-PHE, 3-PHE, and 1-Hydroxypyrene (PYR).

**Table 1:** Participant characteristics.

Characteristics	All participants (N = 3673)	Psoriasis (N = 127)	Non-Psoriasis (N = 3546)	*P* value
Age,(year)	48.28±16.58	47.91±15.19	48.29±16.62	0.871
Gender,n(%)				0.672
male	1812(49.33)	65(51.18)	1747(49.27)	
female	1861(50.67)	62(48.82)	1799(50.73)	
Race,n(%)				**0.016**
Mexican American	441(12.01)	8(6.30)	433(12.21)	
Other Hispanic	293(7.98)	13(10.24)	280(7.90)	
Non-Hispanic White	1746(47.54)	76(59.84)	1670(47.10)	
Non-Hispanic Black	825(22.46)	19(14.96)	806(22.73)	
Other Race	368(10.02)	11(8.66)	357(10.07)	
Education level,n(%)				0.913
Less Than 9th Grade	306(8.33)	13(10.24)	293(8.26)	
9-11th Grade	475(12.93)	18(14.17)	457(12.89)	
High School Grad or Equivalent	775(21.10)	25(19.69)	750(21.15)	
Some College or AA degree	1133(30.85)	37(29.13)	1096(30.91)	
College Graduate or above	984(26.79)	34(26.77)	950(26.79)	
Marital status,n(%)				0.302
unmarried	2774(75.52)	91(71.65)	2683(75.66)	
married	899(24.48)	36(28.35)	863(24.34)	
PIR,n(%)				0.676
0–4.99	2925(79.64)	103(81.10)	2822(79.58)	
≥5	748(20.36)	24(18.90)	724(20.42)	
BMI,kg/m^2^,n(%)				0.277
<24.9	1017(27.69)	29(22.83)	988(27.86)	
24.9–30.0	1267(34.49)	42(33.07)	1225(34.55)	
>30.0	1389(37.82)	56(44.09)	1333(37.59)	
Alcohol status,n(%)				0.802
<12 drinks/year	2683(73.05)	94(3.50)	2589(96.50)	
>12 drinks/year	990(26.95)	33(33.34)	957(66.66)	
Hypertention,n(%)				0.104
yes	1340(36.48)	55(43.31)	1285(26.24)	
no	2333(63.52)	72(56.69)	2261(63.76)	
Diabetes,n(%)				0.125
yes	472(12.85)	22(17.32)	450(12.69)	
no	3201(87.15)	105(82.68)	3096(87.31)	
Hyperlipidemia,n(%)				**0.001**
yes	1377(37.49)	65(51.18)	1312(37.00)	
no	2296(62.51)	62(48.82)	2234(63.00)	
Cotinine, ng/mL	55.01±125.03	69.81±135.25	54.48±124.64	**0.023**
1-NAP,ng/g	3.04±40.864	3.25±20.14	3.03±41.42	0.181
2-NAP,ng/g	0.701±0.979	0.90±1.31	0.69±0.96	**0.012**
2-FLU,ng/g	0.054±0.089	0.07±0.11	0.05±0.09	**0.001**
3-FLU,ng/g	0.026±0.055	0.03±0.05	0.03±0.05	**0.010**
1-PHE,ng/g	0.019±0.029	0.03±0.05	0.02±0.03	**0.032**
2-PHE,ng/g	0.01±0.017	0.01±0.02	0.01±0.02	**0.011**
3-PHE,ng/g	0.013±0.031	0.02±0.03	0.01±0.03	**0.005**
1-PYR,ng/g	0.018±0.04	0.03±0.07	0.02±0.04	**0.002**

PIR, poverty income ratio; BMI, body mass index; NAP, Hydroxynaphthalene; FLU, Hydroxyfluorene; PHE, Hydroxyphenanthrene; PYR, Hydroxypyrene.

### Correlation among urinary PAHs metabolites

**Fig 2:**
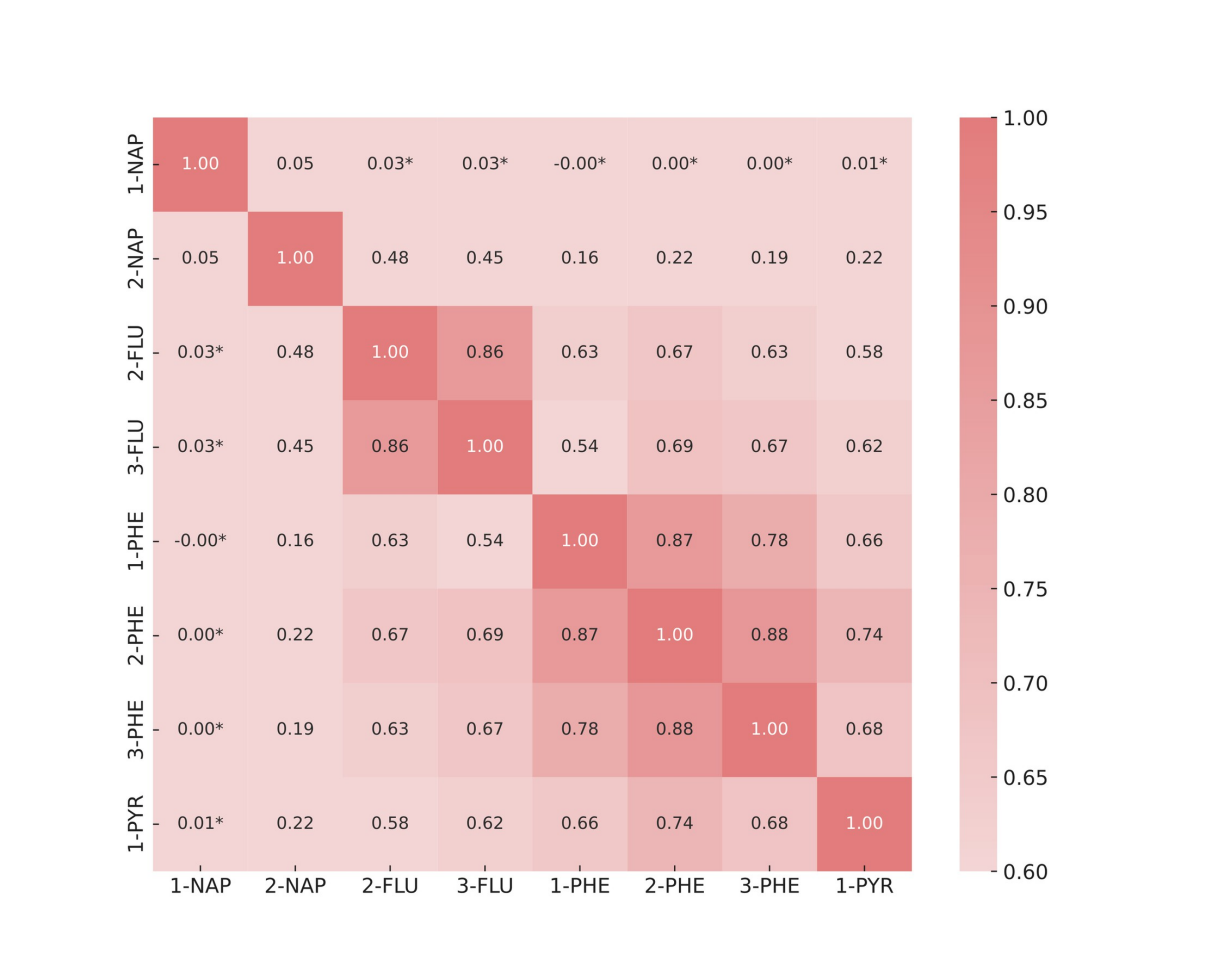
Pearson correlation between urinary PAHs. The connection was statistically significant for all metabolites (*P* < 0.05), with the exception of 1-NAP. *: *P* > 0.05.

The Pearson correlation analysis demonstrated significant associations among all metabolites, except for 1-NAP. Furthermore, the investigation showed a substantial correlation within the FLU and PHE metabolites. This was apparent from the heightened color intensity in their respective data representations, which indicated a more robust and dependable correlation (Fig 2).

### Higher urine PAHs metabolite levels are linked to a greater odds of psoriasis

We utilized multivariate logistic regression modeling to establish the association between concentrations of PAHs and psoriasis (Table 2). After doing calculations on the eight PAHs as continuous variables, we found a significant correlation between all PAH metabolites and the occurrence of psoriasis in crude Model 1, except for 1-NAP which had no link with psoriasis. Model 2 revealed that there was no correlation between 1-PHE and psoriasis. Similarly, the results were stable for model 3 even after considering all covariates. To be precise, when stratified by tertiles, compared to the lowest tertile: tertile 3 for 2-NAP (OR 1.63, 95% CI 1.02, 2.61), tertile 3 for 2-FLU (OR 1.83, 95% CI 1.14, 2.96), tertile 2 (OR 1.65, 95% CI 1.01, 2.69) and tertile 3 (OR 1.85, 95% CI 1.14, 3.00) for 3-PHE showed greater odds of psoriasis prevalence. This implies that when concentrations of these metabolites increase, so does the likelihood of acquiring psoriasis.

**Table 2:** Correlation between specific PAH metabolite and likelihood of developing psoriasis.

PAH		Model 1	Model 2	Model 3
		OR(95%CI),*P* value	OR(95%CI),*P* value	OR(95%CI),*P* value
1-NAP	Tertile 1	1.00	1.00	1.00
	Tertile 2	0.95(0.60,1.49),0.81	0.95(0.60,1.49),0.81	1.02(0.63,1.58),0.99
	Tertile 3	1.23(0.81,1.89),0.33	1.21(0.79,1.86),0.39	1.17(0.75,1.83),0.47
	*P* trend	0.11	0.16	0.22
2-NAP	Tertile 1	1.00	1.00	1.00
	Tertile 2	1.32(0.83,2.11),0.24	1.39(0.87,2.23),0.17	1.33(0.82,2.15),0.23
	Tertile 3	**1.68(1.08,2.63),0.02**	**1.77(1.12.2.77),0.01**	**1.63(1.02,2.61),0.04**
	*P* trend	**<0.01**	**<0.01**	**0.02**
2-FLU	Tertile 1	1.00	1.00	1.00
	Tertile 2	1.46(0.91,2.37),0.12	1.47(0.90,2.38),0.12	1.45(0.89,2.37),0.13
	Tertile 3	**1.97(1.25,3.11),<0.01**	**1.91(1.21,3.03),<0.01**	**1.83(1.14,2.96),0.01**
	*P* trend	**<0.01**	**<0.01**	**<0.01**
3-FLU	Tertile 1	1.00	1.00	1.00
	Tertile 2	1.18(0.74,1.88).0.48	1.16(0.72,1.84),0.54	1.21(0.75,1.93),0.43
	Tertile 3	**1.58(1.02,2.45),0.04**	1.52(0.97,2.37),0.06	1.56(0.93,2.62),0.09
	*P* trend	**<0.01**	**0.02**	**0.03**
1-PHE	Tertile 1	1.00	1.00	1.00
	Tertile 2	**1.60(1.02,2.53),0.04**	1.52(0.96,2.42),0.08	1.46(0.92,2.34),0.11
	Tertile 3	1.54(0.97,2.43),0.06	1.39(0.86,2.23),0.18	1.30(0.78,2.15),0.31
	*P* trend	**0.02**	0.06	0.09
2-PHE	Tertile 1	1.00	1.00	1.00
	Tertile 2	1.21(0.77,1.93)0.41	1.16(0.73,1.84),0.53	1.08(0.67,1.72),0.76
	Tertile 3	1.55(1.00,2.41),0.05	1.43(0.92,2.23),0.11	1.25(0.77,2.02),0.36
	*P* trend	**<0.01**	**<0.01**	**0.03**
3-PHE	Tertile 1	1.00	1.00	1.00
	Tertile 2	**1.73(1.07,2.80),0.02**	**1.70(1.04,2.76),0.03**	**1.65(1.01,2.69),0.04**
	Tertile 3	**2.04(1.28,3.27),<0.01**	**1.92(1.20,3.09),<0.01**	**1.85(1.14,3.00),0.01**
	*P* trend	**<0.01**	**<0.01**	**<0.01**
1-PYR	Tertile 1	1.00	1.00	1.00
	Tertile 2	1.47(0.92,2.34),0.10	1.47(0.92,2.35),0.11	1.46(0.90,2.34),0.12
	Tertile 3	**1.67(1.06,2.63),0.02**	**1.61(1.01,2.56),0.04**	1.50(0.94,2.44),0.08
	*P* trend	**<0.01**	**<0.01**	**<0.01**

Model 1 did not adjust any covariables. Model 2 adjusted for age, sex, and race. Model 3 adjusted for all covariates, including age, sex, race, education level, marital status, household income, PIR, BMI, alcohol use, presence of hypertension, diabetes, hyperlipidemia, and serum cotinine levels. OR, odds ratios; CI, confidence interval.

**Fig 3:**
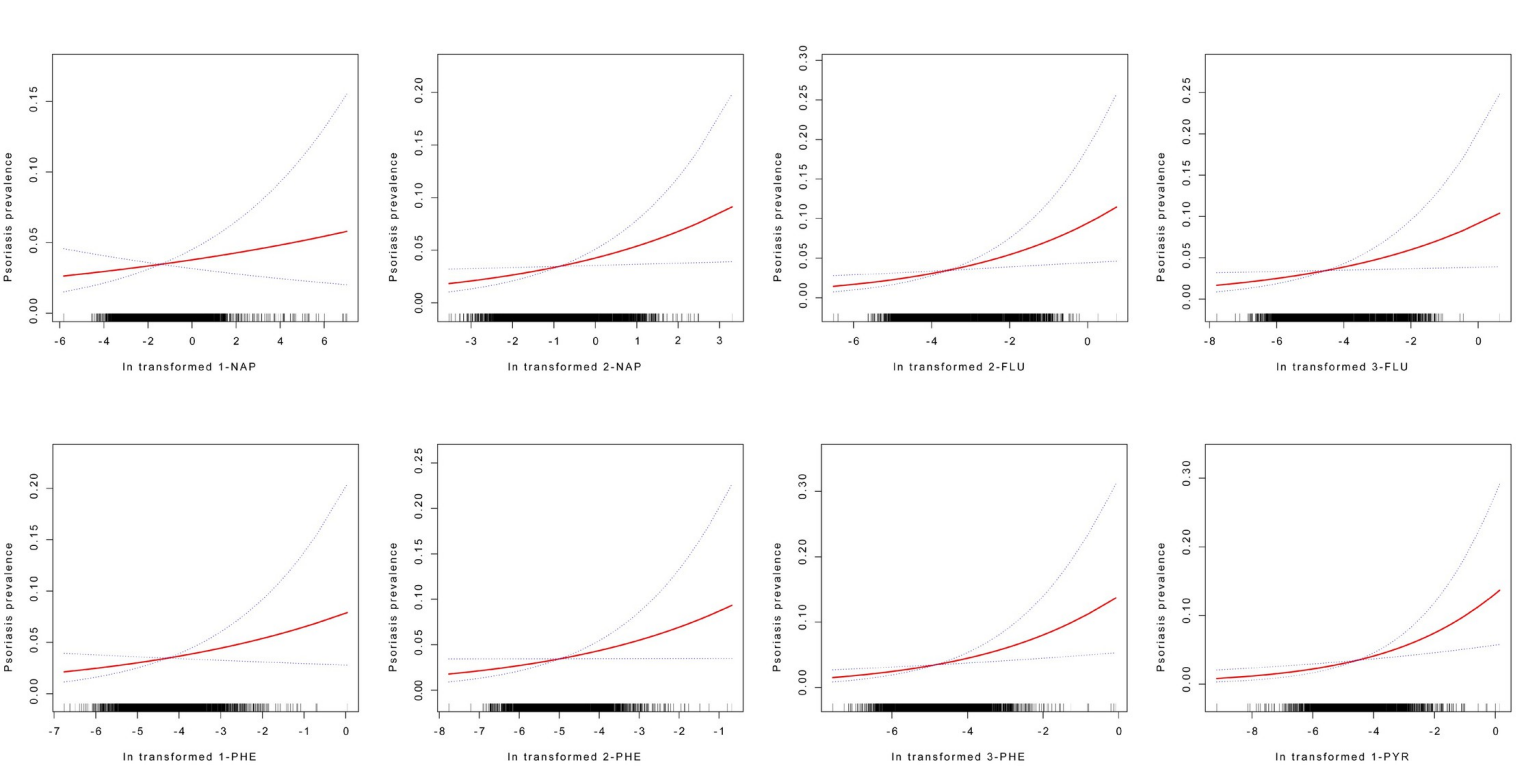
Association between PAHs and the likelihood of developing psoriasis. NAP, Hydroxynaphthalene; FLU, Hydroxyfluorene; PHE, Hydroxyphenanthrene; PYR, Hydroxypyrene. All PAH values were subjected to a log_2_ transformation.

In general, there was a positive correlation between the likelihood of getting psoriasis and urine PAH concentrations, as shown by the smoothed curve fit in Fig 3. This correlation remained significant after accounting for all covariables.

### Subgroup analysis

In order to conduct a more thorough examination of the relationship between urinary PAHs and psoriasis, we conducted subgroup analyses by taking into account multiple factors such as gender, age, race, PIR, BMI, serum cotinine, and hyperlipidemia status. The findings of these analyses are displayed in Table 3. Subgroup analyses demonstrated varying correlations between levels of urine PAH metabolites and psoriasis in different groups. In comparison, participants who were male, aged between 40–60 years, with a BMI >30, and suffered from hyperlipidemia showed a significant correlation between urinary PAH metabolite levels and psoriasis. Nevertheless, the correlation between urinary PAHs and psoriasis did not reveal a noteworthy interaction among the aforementioned variables (*P* interaction > 0.05).

**Table 3:** Subgroup analysis of the association between PAHs and odds of PSO prevalence.

Subgroup	1-NAP	2-NAP	2-FLU	3-FLU	1-PHE	2-PHE	3-PHE	1-PYR
Gender								
male	**1.20(1.00,1.46)**	**1.41(1.05,1.89)**	**1.39(1.04,1.84)**	1.25(0.97,1.61)	1.22(0.86,1.72)	1.34(0.98,1.84)	**1.34(1.01,1.77)**	1.24(0.95,1.61)
female	0.98(0.81,1.19)	1.14(0.83,1.57)	1.37(0.97,1.94)	1.33(0.97,1.82)	1.22(0.84,1.78)	1.24(0.85,1.79)	**1.42(1.02,1.99)**	**1.59(1.17,2.15)**
*P* value	0.07	0.33	0.96	0.77	0.98	0.73	0.77	0.21
Age (years)								
<40	1.18(0.93,1.50)	1.43(0.98,2.08)	1.26(0.84,1.89)	1.20(0.84,1.71)	1.07(0.67,1.70)	1.06(0.68,1.67)	1.31(0.88,1.96)	1.09(0.74,1.62)
40–60	1.07(0.89,1.29)	1.16(0.83,1.63)	**1.51(1.08,2.11)**	**1.36(1.00,1.85)**	1.38(0.94,2.03)	1.40(0.98,2.01)	**1.40(1.02,1.93)**	**1.55(1.17,2.05)**
>60	0.95(0.71,1.26)	1.50(0.99,2.27)	1.39(0.87,2.22)	1.24(0.80,1.92)	1.19(0.71,2.00)	1.50(0.91,2.46)	1.35(0.86,2.09)	1.54(0.99,2.40)
*P* value	0.49	0.57	0.79	0.85	0.69	0.52	0.96	0.22
Race								
Mexican American	0.99(0.74,1.35)	1.19(0.44,3.26)	0.69(0.19,2.53)	0.68(0.23,2.00)	0.87(0.26,2.85)	0.60(0.17,2.12)	0.87(0.29,2.63)	1.01(0.36,2.87)
Other Hispanic	1.26(0.83,1.91)	1.49(0.74,3.00)	1.66(0.80,3.45)	1.86(0.96,3.62)	1.51(0.76,3.02)	1.68(0.81,3.48)	1.62(0.82,3.21)	**2.10(1.02,4.31)**
Non-Hispanic White	1.05(0.89,1.25)	1.25(0.94,1.66)	**1.37(1.03,1.82)**	1.27(0.99,1.64)	1.21(0.86,1.71)	1.21(0.87,1.69)	1.33(0.99,1.77)	**1.41(1.09,1.81)**
Non-Hispanic Black	0.73(0.48,1.13)	1.28(0.75,2.18)	0.74(0.37,1.51)	0.75(0.42,1.35)	0.98(0.47,2.05)	1.23(0.59,2.55)	1.42(0.73,2.75)	1.00(0.56,1.78)
Other Race	1.04(0.60,1.80)	1.31(0.55,3.13)	**2.41(1.11,5.22)**	1.85(0.91,3.76)	1.52(0.65,3.59)	1.61(0.76,3.42)	1.70(0.90,3.22)	0.92(0.39,2.22)
*P* value	0.31	0.99	0.17	0.14	0.86	0.63	0.84	0.43
BMI (kg/m^2^)								
<25	1.23(0.89,1.70)	**1.63(1.01,2.65)**	1.64(0.97,2.77)	1.43(0.87,2.34)	1.09(0.57,2.08)	1.58(0.90,2.77)	1.43(0.84,2.43)	1.44(0.88,2.36)
25–30	1.07(0.86,1.35)	1.30(0.92,1.84)	**1.44(1.09,2.01)**	1.36(0.98,1.87)	1.00(0.62,1.62)	1.10(0.70,1.74)	1.37(0.94,2.00)	1.28(0.90,1.82)
>30	1.05(0.87,1.26)	1.22(0.88,1.69)	1.29(0.91,1.82)	1.18(0.86,1.61)	**1.50(1.05,2.14)**	1.32(0.93,1.87)	**1.43(1.04,1.98)**	**1.50(1.12,2.01)**
*P* value	0.72	0.60	0.74	0.75	0.37	0.61	0.98	0.79
Hyperlipidemia								
yes	1.15(0.98,1.36)	**1.41(1.06,1.87)**	**1.46(1.09,1.97)**	**1.35(1.03,1.78)**	1.28(0.92,1.76)	1.33(0.98,1.82)	**1.46(1.12,1.91)**	**1.47(1.15,1.88)**
no	0.96(0.78,1.17)	1.18(0.89,1.57)	1.23(0.88,1.70)	1.15(0.86,1.54)	1.10(0.76,1.60)	1.22(0.85,1.73)	1.22(0.89,1.69)	1.20(0.89,1.61)
*P* value	0.17	0.35	0.43	0.42	0.55	0.68	0.37	0.27

NAP, Hydroxynaphthalene; FLU, Hydroxyfluorene; PHE, Hydroxyphenanthrene; PYR, Hydroxypyrene. The results are expressed as OR (95% CI). P value here is equivalent to P interaction.

### WQS regression of the associations between urinary PAH metabolite co-exposure and psoriasis

**Fig 4:**
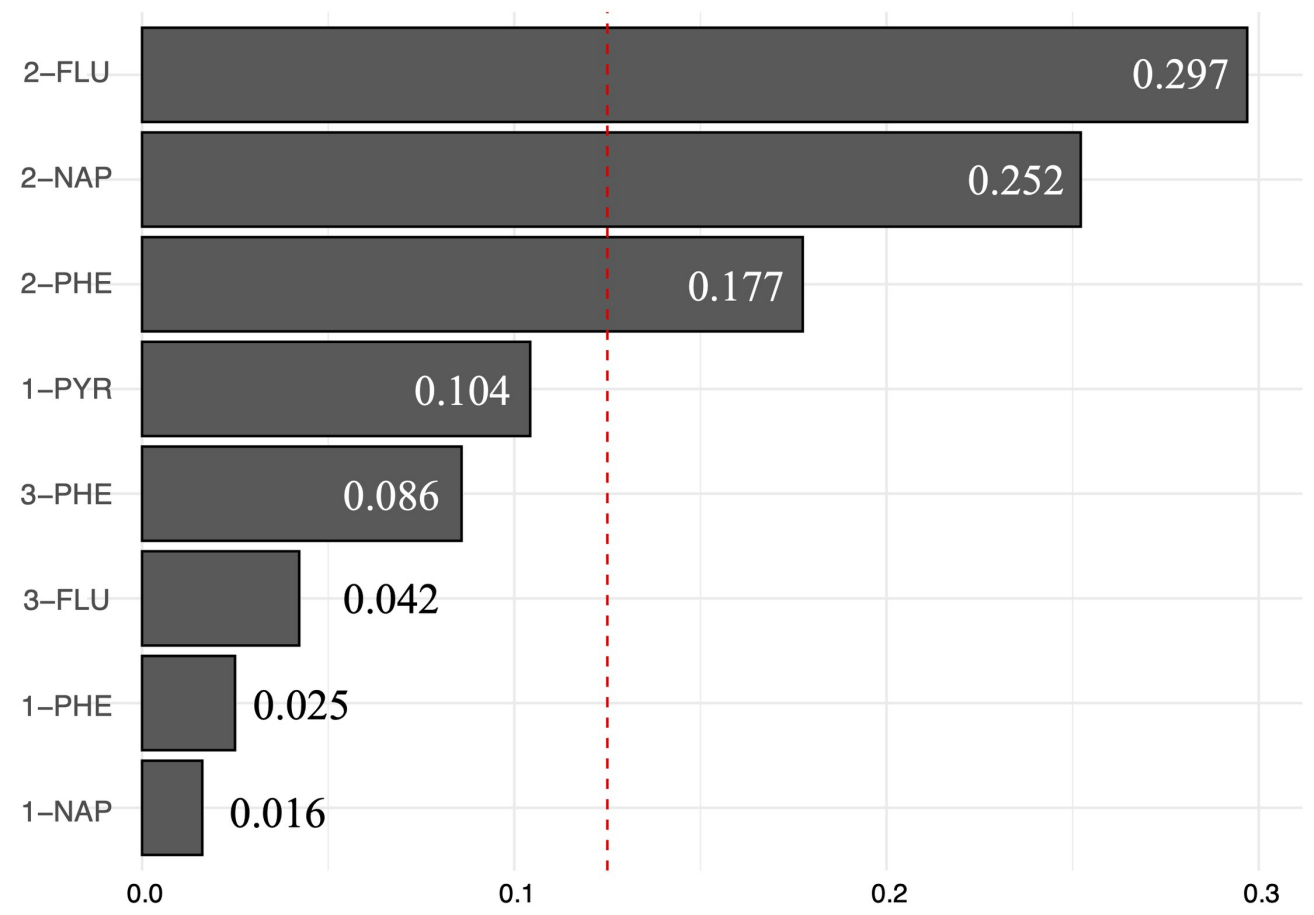
Association between combined urinary PAH metabolites and psoriasis using the WQS model. To minimize weakness in the distribution of concentrations, all urinary PAH metabolites underwent log_2_ transformation. WQS, weighted quantile sum. NAP, Hydroxynaphthalene; FLU, Hydroxyfluorene; PHE, Hydroxyphenanthrene; PYR, Hydroxypyrene.

After accounting for all covariates, the WQS regression analysis revealed a significant positive correlation between the mixture of urine PAH metabolites and psoriasis (OR 1.43, 95% CI 1.11, 1.84). Among the mixes, 2-FLU had the most significant impact on psoriasis with a value of 0.297, followed by 2-NAP at 0.252 and 1-PHE at 0.177. This suggests that these substances have a crucial role in influencing the odds of psoriasis, as seen in Fig 4.

## Discussion

We conducted an extensive correlation study on a sample of 43,481 individuals using the NHANES database. This study examined the correlation between different urinary PAHs and the odds of psoriasis, presenting three findings. An obvious association was established between the presence of PAHs in urine and the occurrence of psoriasis. In addition, through the use of regression analysis, it was determined that there is a substantial and positive correlation between six PAHs and psoriasis, including 2-NAP, 2-FLU, 3-FLU, 2-PHE, 3-PHE, and 1-PYR. Crucially, this correlation remained stable even after considering several covariables, suggesting that these PAHs are linked to a higher likelihood of developing psoriasis. After being divided into three groups based on their levels, it was demonstrated that tertile 3 for 2-NAP, tertile 3 for 2-FLU, and tertiles 2 and 3 for 3-PHE were closely associated with psoriasis prevalence. Thirdly, in subgroup studies, it was found that urine PAH metabolites were strongly correlated with the odds of psoriasis in specific categories. These groups include males, aged 40–60 years, with a BMI > 30, and individuals with hyperlipidemia. The findings of the WQS indicate a direct association between the combinations of urine PAH metabolites and the odds of developing hyperlipidemia, with 2-FLU being the most important contributor.

PAHs are universal environmental pollutants that impose a substantial burden on both individuals and healthcare systems. Overall, there was a clear correlation between PAHs and inflammatory diseases. For instance, Beidelschies et al. performed a study that investigated the association between PAHs and rheumatoid arthritis (RA). The findings indicated that those in the top quartile of single PAHs had a higher likelihood of having RA, and there was a significant link between the physical load of PAHs and developing RA [23]. Wu et al. discovered a direct correlation between periodontitis and the presence of 1-NAP, 2-NAP, 2-FLU, 3-FLU, 1-PHE, and 1-PYR. Additionally, higher levels of PAHs were found to be positively linked with tooth attachment loss, increased periodontal pocket depth, and a greater number of lost teeth [24]. A study conducted by Li et al. found a link between exposure to PAHs and a higher occurrence of metabolic syndrome in both adults and adolescents [25]. Studies in dermatology have also shown a connection between being exposed to air pollutants, particularly PAHs, and the occurrence of inflammatory skin disorders such as atopic dermatitis [26], and acne [27], a study by Guo et al. similarly confirmed the robust correlation between environmental pollutants and psoriasis [28].

The proposed association between PAHs and psoriasis in this study is theoretically plausible. The research on the correlation between air pollution and the progression of psoriasis has primarily focused on the aryl hydrocarbon receptor (AhR) pathway, oxidative stress pathway, genetic pathways, and the subsequent immunological effects. AhR is a transcription factor that requires the presence of specific molecules, known as ligands, to become active. These ligands can come from various sources such as food, microbes, metabolites, and environmental contaminants [29]. The binding of urinary PAH metabolites to AhR can mediate the production of reactive oxygen species (ROS), regulate the expression of noncoding RNAs (e.g., microRNAs), induce cellular differentiation of Th2, Th17, and Th22, and promote the expression of a variety of cytokines or inflammatory factors, resulting in a Th17 immune response and an inflammatory infiltrate that contributes to psoriasis pathogenesis [30]. ROS plays a role in both immune cell receptor signaling and the oxidative stress response [31]. When different signaling pathways, such as mitogen-activated protein kinase (MAPK), Janus tyrosine kinase signal transducer and activator of transcription (JAK-STAT), and nuclear factor-kappa B (NF-κB) pathway, are activated, they contribute to the transcription of cytokines, the inflammatory response, and programmed cell death [32]. Studies have shown that PAHs might increase ROS levels and cause oxidative damage [33]. This process induces the development and specialization of Th17 cells, leading to an increase in the production of IL-17 and IL-22. This, in turn, activates receptors that are associated with macrophages. Consequently, this leads to the proliferation and differentiation of keratinocytes, ultimately facilitating the onset and progression of psoriasis [34, 35]. It has also been proposed that compounds such as PAHs have the ability to alter human epigenetic traits, leading to apoptotic signaling, disruption of the cell cycle, and a response to DNA damage [30]. The main mechanism of this process consists of three separate procedures: DNA methylation [36], histone modification [37], and noncoding RNA [38]. Urinary PAH metabolites may play an essential role in the etiology of psoriasis via these processes, highlighting the potential public health advantages of PAH monitoring and reduction.

Taking into account the fact that these metabolites are exposed to air in a mixture, we evaluated the collective impact of PAH metabolites mixtures on psoriasis using the WQS model. Our findings revealed a clear and positive link between them. The findings indicated that 2-FLU maintained the highest proportion compared to all other mixes, which is consistent with the study by Zhou et al [12]. Prior research has demonstrated that 2-FLU is strongly implicated in the development of cognitive decline [39] and metabolic related fatty liver disease [40]. Nevertheless, the existing studies are insufficient to clarify why 2-FLU has the greatest weight in the association with the risk of psoriasis. We hypothesize that these pathways may be attributed to the immunostimulatory and immunosuppressive effects caused by exposure to PAHs [41]. Nonetheless, our findings suggest a potential preventative method that could reduce the risk of psoriasis to some extent by limiting exposure to certain PAH metabolites.

It is important to highlight that we discovered connections between urine PAH metabolites and psoriasis in certain groups throughout our subgroup analysis. The association between levels of urinary PAH metabolites and psoriasis is stronger in males than in females, likely due to the impact of air pollutants, specifically PAHs, which function as endocrine disrupting chemicals (EDCs). These EDCs disrupt the normal functioning of hormones in males and elevate the levels of pro-inflammatory cytokines such as IL-1b, IL-2, IL-6, IL-10, and IL-17A, leading to immune system disorders [42, 43]. The correlation between urine PAH metabolite levels and psoriasis was consistently stronger in individuals aged 40–60, those with a BMI > 30, and those with hyperlipidemia. This could be attributed to the fact that obesity, as a separate risk factor, results in higher respiratory rates and increased exposure to inhaled air pollutants. This leads to a complex interaction between adipokines and pro-inflammatory cytokines, exacerbating the body’s inflammatory response [44, 45]. Furthermore, due to the lipophilic nature of PAHs, they can easily penetrate the human body through direct contact with the skin and be stored in the fatty tissue of many organs. The accumulation of metabolites and inflammatory factors in adipocytes as a person grows older has a compounding effect. This leads to dyslipidemia and abnormal cellular function, which in turn triggers lipid oxidation and metabolic disorders. These processes ultimately worsen local or systemic inflammatory responses. As a result, individuals who are aged 40–60 years old, obese, and hyperlipidemic are at a higher risk for developing psoriasis [12, 46].

The distinctive advantages of this study include the following. This study utilized a substantial volume of real-world data from several regions nationwide for analysis. Unlike prior studies, we controlled for several covariates in our study and rigorously assessed the influence of social environment and individual health conditions on psoriasis. Furthermore, we examined eight distinct PAHs individually instead of as mixtures and investigated the correlations between various PAHs and the odds of acquiring psoriasis with greater specificity. Furthermore, we conducted Pearson correlation analysis and subgroup analyses to thoroughly evaluate the association between individual and combined PAH metabolites and psoriasis from several angles, therefore enhancing the reliability of the findings.

Nevertheless, it possesses several inherent constraints. Due to the nature of this study being cross-sectional, it was not feasible to conduct a causality analysis. Furthermore, even after accounting for covariates, we were unable to completely eradicate the impact of all risk factors on the computed outcomes. Furthermore, this study relied solely on data from the U.S. population, which restricts the extent to which the findings may be applied to other populations. To validate these conclusions, further data will be required in future research.

## Conclusions

Urinary PAHs metabolite concentrations, including 2-NAP, 2-FLU, 3-FLU, 2-PHE, 3-PHE, and 1-PYR, were found to significantly positively associate with the odds of psoriasis prevalence. Significantly, out of all the compounds, 2-FLU had the greatest impact among the combination of urine PAH metabolites.

Future research could use a longitudinal study design to track individuals’ PAH exposure and health changes over time in order to understand the dynamic link between the two better. Furthermore, mechanistic studies will be required to investigate how PAH influences the development of psoriasis via specific biological pathways. Combining these findings will contribute to a more complete knowledge and a scientific foundation for psoriasis preventive and intervention techniques.

## Supporting information

S1 Dataset (CSV)
